# Association between acquired resistance to PLX4032 (vemurafenib) and ATP-binding cassette transporter expression

**DOI:** 10.1186/1756-0500-7-710

**Published:** 2014-10-10

**Authors:** Martin Michaelis, Florian Rothweiler, Thomas Nerreter, Marijke van Rikxoort, Richard Zehner, Wilhelm G Dirks, Michael Wiese, Jindrich Cinatl

**Affiliations:** Institut für Medizinische Virologie, Klinikum der Goethe-Universität, Paul Ehrlich-Str. 40, Frankfurt am Main, 60596 Germany; Centre for Molecular Processing and School of Biosciences, University of Kent, Canterbury Kent, CT2 7NJ UK; Institut für Rechtsmedizin, Klinikum der Goethe-Universität, Kennedyallee 104, Frankfurt am Main, 60596 Germany; Leibniz-Institute Deutsche Sammlung für Mikroorganismen und Zellkulturen GmbH, Inhoffenstraße 7B, Braunschweig, 38124 Germany; Pharmaceutical Institute, University of Bonn, An der Immenburg 4, Bonn, 53121 Germany; Centre for Molecular Processing and School of Biosciences, University of Kent, Canterbury Kent, CT2 7NJ UK; Institute of Pharmacology and Toxicology, Biomedical Center (BMZ), University of Bonn, Bonn, 53127 Germany

**Keywords:** Vemurafenib, PLX4032, PLX4720, Acquired drug resistance, Melanoma, Mitoxantrone, Vincristine, ABCB1, ABCC1, ABCG2

## Abstract

**Background:**

Various kinase inhibitors are known to be ATP-binding cassette (ABC) transporter substrates and resistance acquisition to kinase inhibitors has been associated to increased ABC transporter expression. Here, we investigated the role of the ABC transporters ABCB1, ABCC1, and ABCG2 during melanoma cell resistance acquisition to the V600-mutant BRAF inhibitors PLX4032 (vemurafenib) and PLX4720. PLX4032 had previously been shown to interfere with ABCB1 and ABCG2. PLX4720 had been demonstrated to interact with ABCB1 but to a lower extent than PLX4032.

**Findings:**

PLX4032 and PLX4720 affected ABCC1- and ABCG2-mediated drug transport in a similar fashion. In a panel of 16 V600E BRAF-mutated melanoma cell lines consisting of four parental cell lines and their sub-lines with acquired resistance to PLX4032, PLX4720, vincristine (cytotoxic ABCB1 and ABCC1 substrate), or mitoxantrone (cytotoxic ABCG2 substrate), we detected enhanced ABC transporter expression in 4/4 cytotoxic ABC transporter substrate-resistant, 3/4 PLX4720-resistant, and 1/4 PLX4032-resistant melanoma cell lines.

**Conclusion:**

PLX4032 has the potential to induce ABC transporter expression but this potential is lower than that of PLX4720 or cytotoxic ABC transporter substrates. Since ABC transporters confer multi-drug resistance, this is of relevance for the design of next-line therapies.

**Electronic supplementary material:**

The online version of this article (doi:10.1186/1756-0500-7-710) contains supplementary material, which is available to authorized users.

## Findings

The oncogenic V600-mutant BRAF inhibitor PLX4032 (vemurafenib) caused improved response and survival rates in V600-mutant BRAF melanoma patients but PLX4032 resistance formation remains inevitable. Resistance mechanisms involve activation of alternative kinases and non-related compensatory pathways [1, 2]. Although protein kinase inhibitors are rather specific drugs (particularly in comparison to the cytotoxic anti-cancer chemotherapeutics) they are also known to exert off-target effects [3–5]. For example, different protein kinase inhibitors interfere with drug transport mediated by various ATP binding cassette (ABC) transporters including ABCB1 (also known as MDR1 or P-glycoprotein), ABCC1 (also known as MRP1), and ABCG2 (also known as BCRP) [3, 4, 6–9].

ABC transporters play important roles in the passage of drugs, xenobiotics, and food constituents through cellular and tissue barriers and consequently in their absorption, distribution, and excretion. Moreover, different ABC transporters are frequently found highly expressed on cancer cells playing an important role in cancer cell chemoresistance [10–12]. Resistance acquisition to kinase inhibitors may be associated with enhanced ABC transporter expression on cancer cells [13–15].

Some information is already available on the effects of PLX4032 and the closely related V600-mutant BRAF inhibitor PLX4720 [16] on ABC transporter function. PLX4032 and PLX4720 both interfere with ABCB1-mediated drug transport [17–19]. PLX4032 was also shown to interact with ABCG2 (also known as BCRP) [17, 18]. ABCG2 expression was suggested to be an acquired resistance mechanism to PLX4032 [20] although data on ABCG2 expression in cells with acquired PLX4032 resistance are missing.

Recently, we had shown that PLX4032 and PLX4720 differed in their effects on ABCB1-mediated drug transport. Despite the structural similarity of these compounds PLX4032 interfered stronger with ABCB1 function than PLX4720 [19]. Here, we 1) compared the effects of PLX4032 and PLX4720 on ABCG2 and ABCC1 and 2) investigated whether resistance acquisition to these compounds may be associated with enhanced ABC transporter expression.

### Effects of PLX4032 and PLX4720 on ABCG2 and ABCC1 function

**Figure 1:**
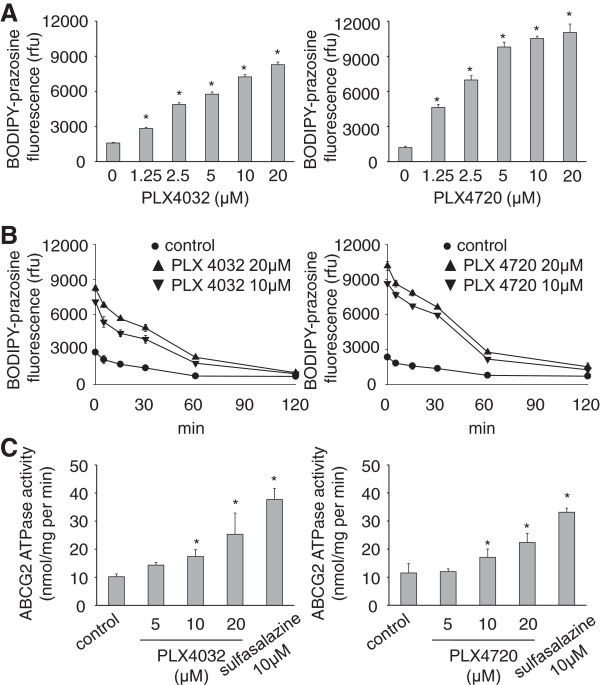
**Effect of PLX4032 and PLX4720 on ABCG2 activity. A)** Influence of PLX4032 or PLX4720 on BODIPY-prazosine (1 μM) fluorescence in UKF-NB-3^ABCG2^ cells, **B)** time kinetics of BODIPY-prazosine (1 μM) fluorescence in UKF-NB-3^ABCG2^ cells in the presence of PLX4032 or PLX4720 after a 60 min pre-incubation period with subsequent wash-out of extracellular BODIPY-prazosine and PLX4032 or PLX4720 (control = BODIPY-prazosine incubation in the absence of drugs). **C)** ABCG2 ATPase activity in isolated membranes in the presence of PLX4032 or PLX4720 (control = activity in the absence of drugs). Sulfasalazine, a known ABCG2 substrate, was used for comparison. *P < 0.05 relative to non-treated controls.

To study the effects of PLX4032 and PLX4720 on ABCG2, an ABCG2-expressing sub-line of the BRAF wild-type neuroblastoma cell line UKF-NB-3 (UKF-NB-3^ABCG2^) was used that had been established by lentiviral transduction with LeGO vectors (http://www.lentigo-vectors.de) as described previously [21, 22]. All experimental procedures were performed as described previously [22]. PLX4032 and PLX4720 treatment of UKF-NB-3^ABCG2^ cells (but not of UKF-NB-3 cells or UKF-NB-3 cells transduced with a control vector) resulted in a similar dose-dependent increase in the cellular accumulation of the fluorescent ABCG2 substrate BODIPY-prazosine (Figure 1A) without affecting ABCG2 expression (Additional file 1: Figure S1).

In wash-out experiments, cellular BODIPY-prazosine fluorescence levels declined rapidly in UKF-NB-3^ABCG2^ cells (Figure 1B), likely because the removal of extracellular PLX4032 and PLX4720 resulted in the rapid ABCG2-mediated efflux of PLX4032/PLX4720 and BODIPY-prazosine. PLX4032 and PLX4720 increased ABCG2 ATPase activity in an isolated membrane assay (Figure 1C) indicating binding and activation of ABCG2 by these compounds. Further, PLX4032 and PLX4720 concentration-dependently enhanced the toxicity of the ABCG2 substrate mitoxantrone in UKF-NB-3^ABCG2^ cells but not of non-transduced UKF-NB-3 cells or UKF-NB-3 cells transduced with a control vector (Additional file 2: Table S1) as indicated by MTT assay after five day incubation. These findings are in accordance with previous studies demonstrating PLX4032 to be an ABCG2 substrate [17, 18, 20].

**Figure 2:**
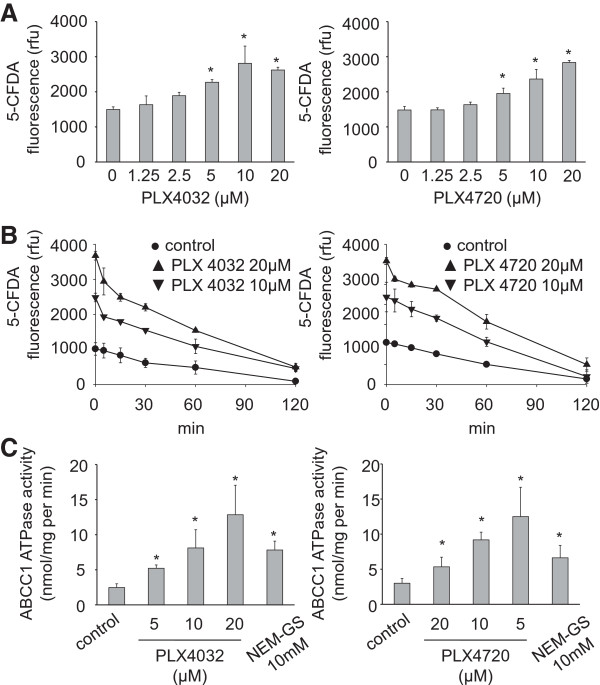
**Effect of PLX4032 and PLX4720 on ABCC1 activity. A)** Influence of PLX4032 or PLX4720 on 5-CFDA (1 μM) fluorescence in G62 cells, **B)** time kinetics of 5-CFDA (1 μM) fluorescence in G62 cells in the presence of PLX4032 or PLX4720 after a 60 min pre-incubation period with subsequent wash-out of extracellular 5-CFDA and PLX4032 or PLX4720 (control = 5-CFDA incubation in the absence of drugs). **C)** ABCC1 ATPase activity in isolated membranes in the presence of PLX4032 or PLX4720 (control = activity in the absence of drugs). NEM-GS, a known ABCC1 substrate, was used for comparison. *P < 0.05 relative to non-treated controls.

The effects of PLX4032 and PLX4720 on ABCC1 were investigated in the ABCC1-expressing cell lines G62 (glioblastoma) and PC3^r^VCR^20^ (prostate cancer) [22]. PLX4032 and PLX4720 similarly increased the accumulation of the fluorescent ABCC1 substrate 5-CFDA in G62 cells in a concentration-dependent manner (Figure 2A) without affecting ABCC1 expression (Additional file 1: Figure S1). 5-CFDA fluorescence declined rapidly after wash-out of PLX4032 or PLX4720 (Figure 2B). Both substances increased ABCC1 ATPase activity in an isolated membrane assay (Figure 2C) and the toxicity of the ABCC1 substrate vincristine in G62 and PC3^r^VCR^20^ cells (Additional file 3: Table S2) in a concentration-dependent manner. These findings appear to contrast other findings that suggested PLX4032 not to be an ABCC1 substrate [20]. The differences may be explained by the use of different ABCC1 substrates and different cellular models. Depending on the exact mode or strength of interaction of ABC transporter modulators with an ABC transporter, different substrates may not always compete for the same binding site. For example, activators of ABCB1 activity that enhance the efflux of certain ABCB1 substrates were described [23]. Moreover, certain ABCB1 modulators were shown to exert differing effects on the cellular accumulation of distinct ABCB1 substrates [24].

PLX4032 and PLX4720 also sensitised two wild-type BRAF melanoma cell lines, IPC-298 and SK-Mel-30 (DSMZ, Braunschweig, Germany), that express ABCB1, ABCC1, and ABCG2 (Additional file 4: Table S3) to vincristine and mitoxantrone (Additional file 5: Table S4). Taken together, these results show that PLX4032 and PLX4720 interfere with ABCC1 and ABCG2 in a similar fashion.

### ABC transporter expression in V600E BRAF-mutated melanoma cell lines with acquired resistance to PLX4032 and PLX4720

**Figure 3:**
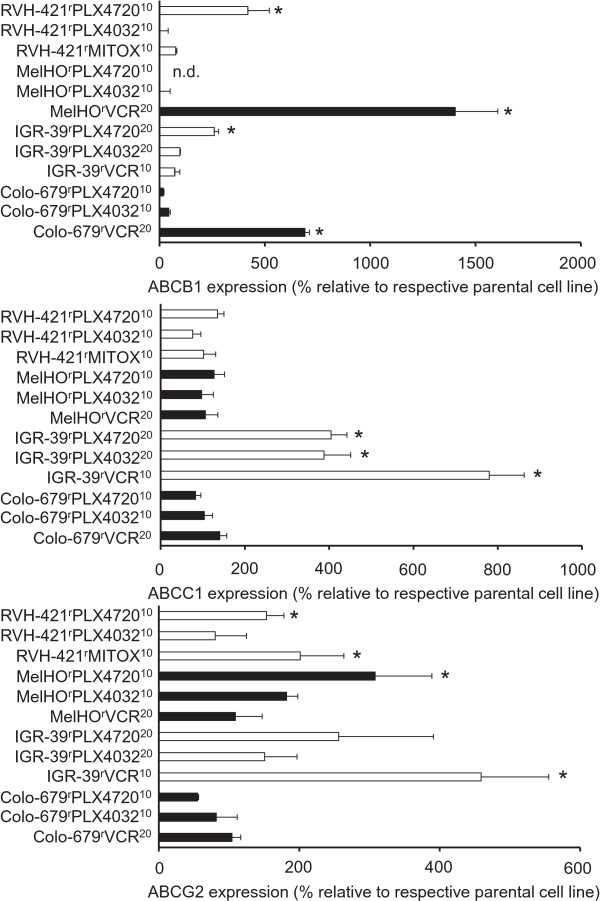
**Expression of ABCB1, ABCC1, and ABCG2 in V600 BRAF-mutated melanoma cells adapted to PLX4032, PLX4720, or the cytotoxic ABC transporter substrates vincristine (VCR) or mitoxantrone (MITOX) relative to the respective parental cell lines.** White and black bars are used to facilitate the identification of resistant sub-lines that are derived from the same parental cell line. *P < 0.05 relative to parental cells.

Next, we investigated the expression of ABCB1, ABCC1, and ABCG2 in the V600E BRAF-mutated melanoma cell lines Colo-679, IGR-39, MelHO, and RVH-421 (DSMZ, Braunschweig, Germany) and their sub-lines with acquired resistance to PLX4032 (Colo-679^r^PLX4032^10 μM^, MelHO^r^PLX4032^10 μM^, IGR-39^r^PLX4032^20 μM^, RVH-421^r^PLX4032^10 μM^), PLX4720 (Colo-679^r^PLX4720^10 μM^, MelHO^r^PLX4720^10 μM^, IGR-39^r^PLX4720^20 μM^, RVH-421^r^PLX4720^10 μM^), vincristine (Colo-679^r^VCR^20^, IGR-39^r^VCR^10^, MelHO^r^VCR^20^), or mitoxantrone (RVH-421^r^Mitox^10^) (Figure 3; Additional file 6: Table S5) that were derived from the Resistant Cancer Cell Line (RCCL) collection (http://www.kent.ac.uk/stms/cmp/RCCL/RCCLabout.html). Treatment of melanoma cells with PLX4032 or cytotoxic anti-cancer drugs had been shown to result in the selection of ABCB5-expressing cells [25]. However, we did not detect enhanced ABCB5 expression in our resistance models relative to the parental sensitive cells.

Colo-679^r^VCR^10^, MelHO^r^VCR^20^, IGR-39^r^PLX4720^20 μM^, and RVH-421^r^PLX4720^10 μM^ expressed increased ABCB1 levels, IGR-39^r^VCR^10^, IGR-39^r^PLX4032^20 μM^, and IGR-39^r^PLX4720^20 μM^ high ABCC1 levels, IGR-39^r^VCR^10^, MelHO^r^PLX4720^10 μM^, RVH-421^r^MITOX^10^, and RVH-421^r^PLX4720^10 μM^ high ABCG2 levels (Figure 3). The ABC transporters conferred resistance to the cytotoxic ABC transporter substrates vincristine (ABCB1, ABCC1) and mitoxantrone (ABCG2) but not to PLX4032 or PLX4720 (Additional file 7: Table S6). These data show that V600-mutant melanoma cells with acquired resistance to PLX4032 may express enhanced ABC transporter levels. However, the potential of PLX4032 to induce ABC transporter expression appears to be lower compared to PLX4720, although both molecules differ in just one phenyl group (Additional file 8: Figure S2), and cytotoxic ABC transporter substrates.

## Discussion

The finding that resistance acquisition to PLX4032 and PLX4720 may be associated with enhanced ABCB1 expression is in line with data showing resistance acquisition to other kinase inhibitors to be associated with ABCB1, ABCC1, and ABCG2 expression [13–15, 26].

PLX4032 had previously been shown to interfere substantially stronger with ABCB1 than PLX4720 [19] while we show here that both compounds display similar effects on ABCG2 and ABCC1. Therefore, differing levels of interaction with the investigated ABC transporters do not explain the enhanced ABC transporter expression in PLX4720-resistant relative to PLX4032-resistant cells. ABC transporter up-regulation may be part of the cellular stress response, and anti-cancer drug-induced stress may induce ABC transporter expression regardless of whether the drugs are ABC transporter substrates [27–29]. PLX4720 induced more pronounced toxic off-target effects (IC_50_ 15.04 ± 0.60 μM in BRAF wild-type UKF-NB-3 cells) than PLX4032 (IC_50_ 30.61 ± 1.37 μM in UKF-NB-3). Also, we detected enhanced ABCG2 expression in IGR-39^r^VCR^10^ cells although vincristine is not regarded to be an ABCG2 substrate.

In conclusion, we show that PLX4032 and PLX4720 interfere with ABCC1- and ABCG2-mediated drug transport. Since ABC transporters play important roles in the absorption, distribution, and excretion of drugs these findings are of potential clinical relevance when PLX4032 is co-administered with anti-cancer drugs or non-cancer-related drugs that are ABC transporter substrates [10–12]. The investigation of a panel of 16 BRAF V600-mutant melanoma cell lines suggested that resistance acquisition to PLX4032 may be associated with enhanced ABC transporter expression although PLX4720 and cytotoxic ABC transporter substrates are stronger inducers of ABC transporter expression than PLX4032. This is of relevance for the design of next-line therapies for melanomas with acquired PLX4032 resistance since ABC transporters including ABCB1, ABCC1, and ABCG2 confer multi-drug resistance [10–12]. The potential clinical implications of our findings are outlined in Additional file 9: Figure S3.

### Availability of supporting data

The data sets supporting the results of this article are included within the article and its additional files.

## Electronic supplementary material

Additional file 1: Figure S1: Effects of PLX4032 and PLX4720 on ABCG2 and ABCC1 expression. A) ABCG2 expression in UKF-NB-3ABCG2 cells after treatment with PLX4032 or PLX4720 for different incubation periods in % relative to non-treated control as determined by flow cytometry; B) ABCC1 expression in G62 cells after treatment with PLX4032 or PLX4720 for different incubation periods in % relative to non-treated control as determined by flow cytometry. (PDF 6 KB)

Additional file 2: Table S1: A. Influence of PLX4032, PLX4720, or the ABCG2 inhibitor fumitremorgin C on the concentration of the ABCG2 substrate mitoxantrone that decreases the viability of ABCG2-expressing UKF-NB-3^ABCG2^ cells by 50% (IC50). B. Influence of PLX4032 or the ABCG2 inhibitor fumitremorgin C on the concentration of the ABCG2 substrate mitoxantrone that decreases the viability of UKF-NB-3 cells by 50% (IC50). C. Influence of PLX4720 or the ABCG2 inhibitor fumitremorgin C on the concentration of the ABCG2 substrate mitoxantrone that decreases the viability of UKF-NB-3 cells by 50% (IC50). D. Influence of PLX4032 or the ABCG2 inhibitor fumitremorgin C on the concentration of the ABCG2 substrate mitoxantrone that decreases the viability of UKF-NB-3 cells transduced with an empty control vector (as comparison to UKF-NB-3ABCG2) by 50% (IC50). E. Influence of PLX4720 or the ABCG2 inhibitor fumitremorgin C on the concentration of the ABCG2 substrate mitoxantrone that decreases the viability of UKF-NB-3 cells transduced with an empty control vector (as comparison to UKF-NB-3ABCG2) by 50% (IC50). (PDF 15 KB)

Additional file 3: Table S2: A. Influence of PLX4032, PLX4720, or the ABCC1 inhibitor MK571 on the concentration of the ABCC1 substrate vincristine that decreases the viability of ABCC1-expressing G62 cells by 50% (IC50). B. Influence of PLX4032 or the ABCC1 inhibitor MK571 on the concentration of the ABCC1 substrate vincristine that decreases the viability of ABCC1-expressing PC3rVCR20 cells by 50% (IC50). C. Influence of PLX4720 or the ABCC1 inhibitor MK571 on the concentration of the ABCC1 substrate vincristine that decreases the viability of ABCC1-expressing PC3rVCR20 cells by 50% (IC50). (PDF 10 KB)

Additional file 4: Table S3: A. ABCB1 expression in melanoma cells and the influence of the ABCB1 inhibitor PGP4008 (2.5 μM) on the concentration of the ABCB1 substrate vincristine that decreases the viability of melanoma cells by 50% (IC50). B. ABCC1 expression in melanoma cells and the influence of the ABCC1 inhibitor MK571 (10 μM) on the concentration of the ABCC1 substrate vincristine that decreases the viability of melanoma cells by 50% (IC50). C. ABCG2 expression in melanoma cells and the influence of the ABCG2 inhibitor fumitremorgin C (10 μM) on the concentration of the ABCG2 substrate mitoxantrone that decreases the viability of melanoma cells by 50% (IC50). (PDF 11 KB)

Additional file 5: Table S4: Influence of PLX4032 (20 μM) or PLX4720 (20 μM) on the vincristine (substrate of ABCB1 and ABCC1) or mitoxantrone (substrate of ABCG2) concentrations that reduce the cell viability by 50% (IC50) in the BRAF wild-type melanoma cell lines IPC298 and SK-MEL-30 that both express ABCB1, ABCC1, and ABCG2. (PDF 6 KB)

Additional file 6: Table S5: Drug concentrations that decrease the viability of V600E BRAFmutated melanoma cells by 50% (IC50). (PDF 6 KB)

Additional file 7: Table S6: A. Influence of the ABCB1 inhibitor verapamil (10 μM) on the sensitivity of ABCB1-expressing melanoma cells to PLX4720 or the cytotoxic ABCB1 substrate vincristine. Concentrations that reduce the cell viability by 50% (IC50) were determined after a five day incubation period by MTT assay. B. Influence of the ABCC1 inhibitor verapamil (10 μM) on the sensitivity of ABCC1-expressing melanoma cells to PLX4032, PLX4720, or the cytotoxic ABCC1 substrate vincristine. Concentrations that reduce the cell viability by 50% (IC50) were determined after a five day incubation period by MTT assay. C. Influence of ABCG2 inhibitor fumitremorgin C (2.5 μM) on the sensitivity of ABCG2-expressing melanoma cells to PLX4720 or the cytotoxic ABCG2 substrate mitoxantrone. Concentrations that reduce the cell viability by 50% (IC50) were determined after a five day incubation period by MTT assay. (PDF 14 KB)

Additional file 8: Figure S2: Chemical structures of PLX4032 and PLX4720. (PDF 36 KB)

Additional file 9: Figure S3: Potential clinical implications of the findings presented in this report. A) PLX4032 and PLX4720 interfere with ABCB1, ABCC1, and ABCG2 function. Since these ABC transporters are expressed at physiological barriers and control the absorption, body distribution, and excretion of their substrates, inhibition of these transporters may modify the pharmacokinetics of co-administered ABC transporter substrates including anti-cancer drugs and drugs that are administered for alternative indications (e.g. antihypertensive drugs). In addition, PLX4032 and PLX4720 may sensitise ABC transporter-expressing cancer cells to co-administered anti-cancer drugs that are ABC transporter substrates. B) Resistance formation to PLX4032 and PLX4720 may be associated with enhanced ABC transporter expression in cancer cells. This enhanced ABC transporter expression will result in cross-resistance of the PLX4032- or PLX4720-resistant cells to anti-cancer drugs that are substrates of the respective ABC transporters. (PDF 6 KB)
