# Supplementary material for: Association between acquired resistance to PLX4032 (vemurafenib) and ATP-binding cassette transporter expression
Source: BMC Res Notes. 2014 Oct 10;7:710. doi: 10.1186/1756-0500-7-710 (PMC4197243; doi:10.1186/1756-0500-7-710)
Supplement: Supplementary file 1 — Additional file 1: Figure S1: Effects of PLX4032 and PLX4720 on ABCG2 and ABCC1 expression. A) ABCG2 expression in UKF-NB-3ABCG2 cells after treatment with PLX4032 or PLX4720 for different incubation periods in % relative to non-treated control as determined by flow cytometry; B) ABCC1 expression in G62 cells after treatment with PLX4032 or PLX4720 for different incubation periods in % relative to non-treated control as determined by flow cytometry. (PDF 6 KB) [file 13104_2014_3224_MOESM1_ESM.pdf]

Suppl. Figure 1

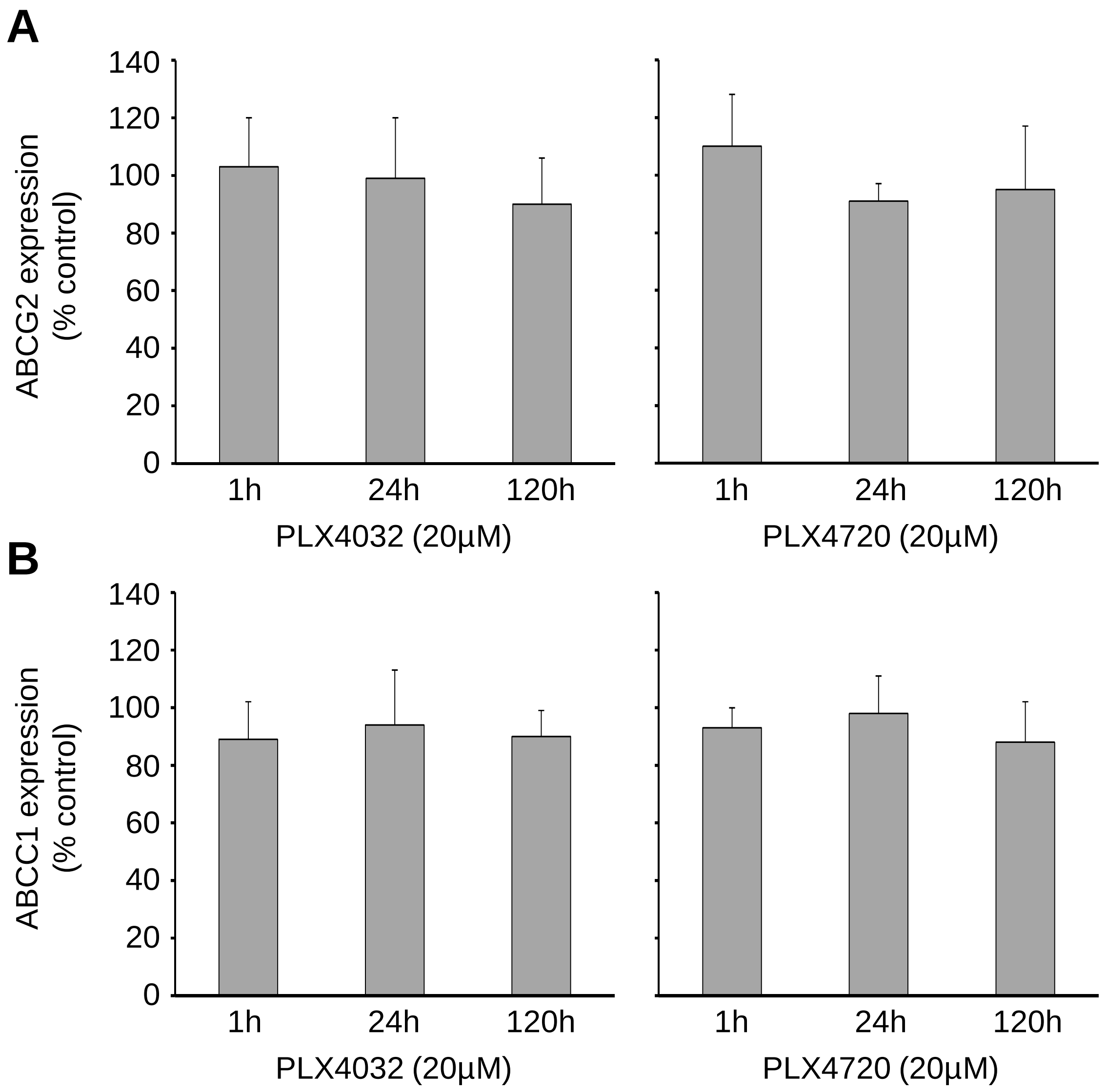

Suppl. Table 1.Effects of PLX4032 and PLX4720 on ABCG2 and ABCC1 expression. A) ABCG2 expression in UKF-NB-3^{ABCG2} cells after treatment with PLX4032 or PLX4720 for different incubation periods in % relative to non-treated control as determined by flow cytometry; B) ABCC1 expression in G62 cells after treatment with PLX4032 or PLX4720 for different incubation periods in % relative to non-treated control as determined by flow cytometry.
