# Supplementary material for: Association between acquired resistance to PLX4032 (vemurafenib) and ATP-binding cassette transporter expression
Source: BMC Res Notes. 2014 Oct 10;7:710. doi: 10.1186/1756-0500-7-710 (PMC4197243; doi:10.1186/1756-0500-7-710)
Supplement: Supplementary file 2 — Additional file 2: Table S1: A. Influence of PLX4032, PLX4720, or the ABCG2 inhibitor fumitremorgin C on the concentration of the ABCG2 substrate mitoxantrone that decreases the viability of ABCG2-expressing UKF-NB-3ABCG2 cells by 50% (IC50). B. Influence of PLX4032 or the ABCG2 inhibitor fumitremorgin C on the concentration of the ABCG2 substrate mitoxantrone that decreases the viability of UKF-NB-3 cells by 50% (IC50). C. Influence of PLX4720 or the ABCG2 inhibitor fumitremorgin C on the concentration of the ABCG2 substrate mitoxantrone that decreases the viability of UKF-NB-3 cells by 50% (IC50). D. Influence of PLX4032 or the ABCG2 inhibitor fumitremorgin C on the concentration of the ABCG2 substrate mitoxantrone that decreases the viability of UKF-NB-3 cells transduced with an empty control vector (as comparison to UKF-NB-3ABCG2) by 50% (IC50). E. Influence of PLX4720 or the ABCG2 inhibitor fumitremorgin C on the concentration of the ABCG2 substrate mitoxantrone that decreases the viability of UKF-NB-3 cells transduced with an empty control vector (as comparison to UKF-NB-3ABCG2) by 50% (IC50). (PDF 15 KB) [file 13104_2014_3224_MOESM2_ESM.pdf]

Suppl. Table 1A. Influence of PLX4032, PLX4720, or the ABCG2 inhibitor fumitremorgin C on the concentration of the ABCG2 substrate mitoxantrone that decreases the viability of ABCG2-expressing UKF-NB-3^{ABCG2} cells by 50% (IC_{50}).

	**cell viability in the absence of mitoxantrone (%)**	**IC_{50} mitoxantrone (ng/mL)**	**fold sensitisation relative to mitoxantrone alone**
**PLX4032 (µM)**			
0	100.00 ± 0.00	42.24 ± 8.94	1.00
0.625	90.34 ± 3.43	43.05 ± 8.24	0.98
1.25	88.62 ± 6.64	32.57 ± 11.09	1.30
2.5	93.69 ± 7.92	7.42 ± 1.82*	5.69
5	81.83 ± 12.65	0.97 ± 0.02*	43.55
10	87.06 ± 11.90	0.48 ± 0.05*	88.00
20	60.56 ± 5.03	0.39 ± 0.12*	108.31
fumitremorgin C (10µM)	77.44 ± 7.14	0.12 ± 0.04*	352.00
**PLX4720 (µM)**			
0	100.00 ± 0.00	42.05 ± 10.35	1.00
0.625	82.39 ± 16.17	26.47 ± 1.35*	1.59
1.25	93.05 ± 8.22	3.61 ± 1.80*	11.65
2.5	88.62 ± 6.00	1.38 ± 0.63*	30.47
5	84.91 ± 7.90	0.59 ± 0.05*	71.27
10	63.61 ± 4.99	0.35 ± 0.04*	120.14
20	46.20 ± 2.20	0.29 ± 0.02*	145.00
fumitremorgin C (10µM)	69.95 ± 4.30	0.13 ± 0.03*	323.46

* $p < 0.05$ relative to mitoxantrone alone

Suppl. Table 1B. Influence of PLX4032 or the ABCG2 inhibitor fumitremorgin C on the concentration of the ABCG2 substrate mitoxantrone that decreases the viability of UKF-NB-3 cells by 50% (IC_{50}).

PLX4032 (µM)	cell viability in the absence of mitoxantrone (%)	IC_{50} mitoxantrone (ng/mL)	fold sensitisation relative to mitoxantrone alone
0	100.00 ± 0.00	0.15 ± 0.06	1.00
0.625	98.05 ± 13.44	0.13 ± 0.04	1.15
1.25	90.86 ± 10.91	0.16 ± 0.05	0.94
2.5	102.50 ± 12.12	0.17 ± 0.07	0.88
5	86.77 ± 10.71	0.12 ± 0.06	1.25
10	71.27 ± 8.01	0.13 ± 0.07	1.15
20	59.98 ± 10.55	0.14 ± 0.03	1.07
fumitremorgin C (µM)			
10	91.30 ± 13.44	0.14 ± 0.04	1.07

Suppl. Table 1C. Influence of PLX4720 or the ABCG2 inhibitor fumitremorgin C on the concentration of the ABCG2 substrate mitoxantrone that decreases the viability of UKF-NB-3 cells by 50% (IC_{50}).

PLX4720 (µM)	cell viability in the absence of mitoxantrone (%)	IC_{50} mitoxantrone (ng/mL)	fold sensitisation relative to mitoxantrone alone
0	100.00 ± 0.00	0.17 ± 0.08	1.00
0.625	94.58 ± 12.24	0.19 ± 0.05	0.89
1.25	88.62 ± 11.84	0.14 ± 0.06	1.21
2.5	92.68 ± 13.32	0.15 ± 0.04	1.13
5	79.22 ± 12.53	0.19 ± 0.03	0.89
10	63.16 ± 15.05	0.15 ± 0.05	1.13
20	43.49 ± 8.99	0.15 ± 0.04	1.13
fumitremorgin C (µM)			
10	88.57 ± 11.64	0.16 ± 0.05	1.06

Suppl. Table 1D. Influence of PLX4032 or the ABCG2 inhibitor fumitremorgin C on the concentration of the ABCG2 substrate mitoxantrone that decreases the viability of UKF-NB-3 cells transduced with an empty control vector (as comparison to UKF-NB-3^{ABCG2}) by 50% (IC_{50}).

PLX4032 (µM)	cell viability in the absence of mitoxantrone (%)	IC_{50} mitoxantrone (ng/mL)	fold sensitisation relative to mitoxantrone alone
0	100.00 ± 0.00	0.22 ± 0.07	1.00
0.625	97.22 ± 11.51	0.23 ± 0.03	0.96
1.25	99.12 ± 9.51	0.24 ± 0.07	0.92
2.5	95.48 ± 10.02	0.20 ± 0.06	1.10
5	89.01 ± 13.00	0.22 ± 0.03	1.00
10	78.61 ± 9.03	0.19 ± 0.05	1.16
20	50.13 ± 9.86	0.20 ± 0.04	1.10
fumitremorgin C (µM)			
10	86.25 ± 9.25	0.20 ± 0.05	1.10

Suppl. Table 1E. Influence of PLX4720 or the ABCG2 inhibitor fumitremorgin C on the concentration of the ABCG2 substrate mitoxantrone that decreases the viability of UKF-NB-3 cells transduced with an empty control vector (as comparison to UKF-NB-3^{ABCG2}) by 50% (IC_{50}).

PLX4720 (µM)	cell viability in the absence of mitoxantrone (%)	IC_{50} mitoxantrone (ng/mL)	fold sensitisation relative to mitoxantrone alone
0	100.00 ± 0.00	0.20 ± 0.05	1.00
0.625	103.93 ± 9.84	0.18 ± 0.04	1.11
1.25	97.14 ± 14.22	0.22 ± 0.04	0.91
2.5	102.21 ± 7.26	0.19 ± 0.05	1.05
5	89.41 ± 13.37	0.17 ± 0.08	1.18
10	67.18 ± 8.62	0.19 ± 0.06	1.05
20	45.52 ± 11.08	0.19 ± 0.05	1.05
fumitremorgin C (µM)			
10	87.49 ± 8.52	0.18 ± 0.06	1.11
