# Supplementary material for: Association between acquired resistance to PLX4032 (vemurafenib) and ATP-binding cassette transporter expression
Source: BMC Res Notes. 2014 Oct 10;7:710. doi: 10.1186/1756-0500-7-710 (PMC4197243; doi:10.1186/1756-0500-7-710)
Supplement: Supplementary file 3 — Additional file 3: Table S2: A. Influence of PLX4032, PLX4720, or the ABCC1 inhibitor MK571 on the concentration of the ABCC1 substrate vincristine that decreases the viability of ABCC1-expressing G62 cells by 50% (IC50). B. Influence of PLX4032 or the ABCC1 inhibitor MK571 on the concentration of the ABCC1 substrate vincristine that decreases the viability of ABCC1-expressing PC3rVCR20 cells by 50% (IC50). C. Influence of PLX4720 or the ABCC1 inhibitor MK571 on the concentration of the ABCC1 substrate vincristine that decreases the viability of ABCC1-expressing PC3rVCR20 cells by 50% (IC50). (PDF 10 KB) [file 13104_2014_3224_MOESM3_ESM.pdf]

Suppl. Table 2A. Influence of PLX4032, PLX4720, or the ABCC1 inhibitor MK571 on the concentration of the ABCC1 substrate vincristine that decreases the viability of ABCC1-expressing G62 cells by 50% (IC_{50}).

	**cell viability in the absence of vincristine (%)**	**IC_{50} vincristine (ng/mL)**	**fold sensitisation relative to vincristine alone**
**PLX4032 (µM)**			
0	100.00 ± 0.00	2.24 ± 0.31	1.00
0.625	99.81 ± 16.78	2.13 ± 0.22	1.05
1.25	104.91 ± 11.93	2.01 ± 0.07	1.11
2.5	95.04 ± 13.73	1.78 ± 0.20	1.26
5	96.44 ± 7.48	1.23 ± 0.14*	1.82
10	92.36 ± 9.95	0.72 ± 0.09*	3.11
20	85.12 ± 14.52	0.55 ± 0.16*	4.07
MK571 10µM	96.79 ± 10.17	0.39 ± 0.13*	5.74
**PLX4720 (µM)**			
0	100.00 ± 0.00	2.18 ± 0.24	1.00
0.625	100.84 ± 18.81	2.19 ± 0.27	1.00
1.25	92.10 ± 10.75	1.97 ± 0.12	1.11
2.5	93.59 ± 12.28	1.64 ± 0.29	1.33
5	87.33 ± 13.18	1.43 ± 0.30*	1.52
10	73.91 ± 12.01	0.75 ± 0.15*	2.91
20	60.53 ± 9.19	0.53 ± 0.15*	4.11
MK571 10µM	95.38 ± 12.35	0.37 ± 0.11*	5.89

* $p < 0.05$ relative to vincristine alone

Suppl. Table 2B. Influence of PLX4032 or the ABCC1 inhibitor MK571 on the concentration of the ABCC1 substrate vincristine that decreases the viability of ABCC1-expressing $PC3^{r}VCR^{20}$ cells by 50% (IC_{50}).

PLX4032 (µM)	cell viability in the absence of vincristine (%)	IC_{50} vincristine (ng/mL)	fold sensitisation relative to vincristine alone
0	100.00 ± 0.00	39.95 ± 3.47	1.00
0.625	93.84 ± 12.04	35.09 ± 6.39	1.14
1.25	95.05 ± 9.57	32.33 ± 4.91	1.24
2.5	96.15 ± 10.35	29.70 ± 4.88	1.35
5	84.06 ± 4.16	17.36 ± 2.23*	2.30
10	88.24 ± 2.41	12.22 ± 1.74*	3.27
20	81.93 ± 2.46	9.29 ± 1.33*	4.30
MK571 (µM)			
10	109.38 ± 16.31	11.87 ± 2.67*	3.37

* $p < 0.05$ relative to vincristine alone

Suppl. Table 2C. Influence of PLX4720 or the ABCC1 inhibitor MK571 on the concentration of the ABCC1 substrate vincristine that decreases the viability of ABCC1-expressing PC3^{r}VCR^{20} cells by 50% (IC_{50}).

PLX4720 (µM)	cell viability in the absence of vincristine (%)	IC_{50} vincristine (ng/mL)	fold sensitisation relative to vincristine alone
0	100.00 ± 0.00	37.29 ± 3.08	1.00
0.625	99.61 ± 10.44	35.30 ± 4.99	1.06
1.25	96.37 ± 8.83	37.55 ± 2.41	0.99
2.5	94.90 ± 8.83	27.67 ± 5.13	1.35
5	97.74 ± 11.32	13.84 ± 2.32*	2.69
10	88.35 ± 15.56	11.15 ± 1.93*	3.34
20	72.85 ± 10.06	9.40 ± 1.08*	3.97
MK571 (µM)			
10	114.96 ± 10.37	11.57 ± 1.88*	3.22

* $p < 0.05$ relative to vincristine alone
