# Supplementary material for: Association between acquired resistance to PLX4032 (vemurafenib) and ATP-binding cassette transporter expression
Source: BMC Res Notes. 2014 Oct 10;7:710. doi: 10.1186/1756-0500-7-710 (PMC4197243; doi:10.1186/1756-0500-7-710)
Supplement: Supplementary file 4 — Additional file 4: Table S3: A. ABCB1 expression in melanoma cells and the influence of the ABCB1 inhibitor PGP4008 (2.5 μM) on the concentration of the ABCB1 substrate vincristine that decreases the viability of melanoma cells by 50% (IC50). B. ABCC1 expression in melanoma cells and the influence of the ABCC1 inhibitor MK571 (10 μM) on the concentration of the ABCC1 substrate vincristine that decreases the viability of melanoma cells by 50% (IC50). C. ABCG2 expression in melanoma cells and the influence of the ABCG2 inhibitor fumitremorgin C (10 μM) on the concentration of the ABCG2 substrate mitoxantrone that decreases the viability of melanoma cells by 50% (IC50). (PDF 11 KB) [file 13104_2014_3224_MOESM4_ESM.pdf]

Suppl. Table 3A. ABCB1 expression in melanoma cells and the influence of the ABCB1 inhibitor PGP4008 (2.5µM) on the concentration of the ABCB1 substrate vincristine that decreases the viability of melanoma cells by 50% (IC_{50}).

		ABCB1 expression		IC_{50} vincristine (ng/ml)		
Cell line	BRAF status		RFU[1]		+ PGP4008	fold sensitisation
IPC298	wild-type	yes	27 ± 9	2.74 ± 0.39	0.75 ± 0.42[2] (79 ± 3)[3]	3.65
SK-MEL-30	wild-type	yes	88 ± 15	4.57 ± 1.49	0.92 ± 0.33[2] (91 ± 10)	4.99

[1] RFU, relative fluorescence units
[2] $P < 0.05$ relative to vincristine alone
[3] cell viability (% non-treated control) in the presence of PGP4008 alone

Suppl. Table 3B. ABCC1 expression in melanoma cells and the influence of the ABCC1 inhibitor MK571 (10 µM) on the concentration of the ABCC1 substrate vincristine that decreases the viability of melanoma cells by 50% (IC_{50}).

		ABCC1 expression		IC_{50} vincristine (ng/ml)		
Cell line	BRAF status		RFU[1]		+ MK571	fold sensitisation
IPC298	wild-type	yes	476 ± 46	2.74 ± 0.39	1.09 ± 0.39[2] (92 ± 16)[3]	2.52
SK-MEL-30	wild-type	yes	1656 ± 133	4.57 ± 1.49	2.59 ± 0.55[2] (98 ± 15)	1.76

[1] RFU, relative fluorescence units
[2] $P < 0.05$ relative to vincristine alone
[3] cell viability (% non-treated control) in the presence of MK571 alone

Suppl. Table 3C. ABCG2 expression in melanoma cells and the influence of the ABCG2 inhibitor fumitremorgin C (10 µM) on the concentration of the ABCG2 substrate mitoxantrone that decreases the viability of melanoma cells by 50% (IC_{50}).

		ABCG2 expression		IC_{50} mitoxantrone (ng/ml)		
Cell line	BRAF status		RFU[1]		+ fumitremorgin C	fold sensitisation
IPC298	wild-type	yes	175 ± 14	1.54 ± 0.45	0.96 ± 0.19[2] (83 ± 9)[3]	1.61
SK-MEL-30	wild-type	yes	68 ± 6	3.44 ± 0.44	2.21 ± 0.34[2] (80 ± 8)	1.56

[1] RFU, relative fluorescence units
[2] P < 0.05 relative to mitoxantrone alone
[3] cell viability (% non-treated control) in the presence of fumitremorgin C alone
