# Supplementary material for: Association between acquired resistance to PLX4032 (vemurafenib) and ATP-binding cassette transporter expression
Source: BMC Res Notes. 2014 Oct 10;7:710. doi: 10.1186/1756-0500-7-710 (PMC4197243; doi:10.1186/1756-0500-7-710)
Supplement: Supplementary file 5 — Additional file 5: Table S4: Influence of PLX4032 (20 μM) or PLX4720 (20 μM) on the vincristine (substrate of ABCB1 and ABCC1) or mitoxantrone (substrate of ABCG2) concentrations that reduce the cell viability by 50% (IC50) in the BRAF wild-type melanoma cell lines IPC298 and SK-MEL-30 that both express ABCB1, ABCC1, and ABCG2. (PDF 6 KB) [file 13104_2014_3224_MOESM5_ESM.pdf]

Suppl. Table 4. Influence of PLX4032 (20µM) or PLX4720 (20µM) on the vincristine (substrate of ABCB1 and ABCC1) or mitoxantrone (substrate of ABCG2) concentrations that reduce the cell viability by 50% (IC_{50}) in the BRAF wild-type melanoma cell lines IPC298 and SK-MEL-30 that both express ABCB1, ABCC1, and ABCG2.

	IC_{50} vincristine (ng/ml)	IC_{50} vincristine in the presence of PLX4032/PLX4720	fold sensitisation[1]	IC_{50} mitoxantrone (ng/mL)	IC_{50} mitoxantrone in the presence of PLX4032/PLX4720	fold sensitisation[2]
**PLX4032**						
IPC298	2.74 ± 0.39	0.54 ± 0.07[3] (73 ± 7)[4]	5.11	1.54 ± 0.45	0.79 ± 0.08[6] (75 ± 8)[4]	1.96
SK-MEL-30	4.57 ± 1.49	0.42 ± 0.07[3] (85 ± 9)[4]	10.82	3.44 ± 0.44	1.73 ± 0.62[6] (79 ± 11)[4]	1.99
**PLX4720**						
IPC298	2.74 ± 0.39	0.83 ± 0.08[3] (57 ± 10)[5]	3.32	1.54 ± 0.45	0.69 ± 0.25[6] (71 ± 13)[5]	2.25
SK-MEL-30	4.57 ± 1.49	1.50 ± 0.62[3] (73 ± 5)[5]	3.04	3.44 ± 0.44	1.61 ± 0.08[6] (77 ± 9)[5]	2.14

[1] relative to vincristine alone
[2] relative to mitoxantrone alone
[3] $p < 0.05$ relative to vincristine alone
[4] cell viability in the presence of PLX4032 alone
[5] cell viability in the presence of PLX4720 alone
[6] $p < 0.05$ relative to mitoxantrone alone
