# Supplementary material for: Association between acquired resistance to PLX4032 (vemurafenib) and ATP-binding cassette transporter expression
Source: BMC Res Notes. 2014 Oct 10;7:710. doi: 10.1186/1756-0500-7-710 (PMC4197243; doi:10.1186/1756-0500-7-710)
Supplement: Supplementary file 6 — Additional file 6: Table S5: Drug concentrations that decrease the viability of V600E BRAFmutated melanoma cells by 50% (IC50). (PDF 6 KB) [file 13104_2014_3224_MOESM6_ESM.pdf]

Suppl. Table 5. Drug concentrations that decrease the viability of V600E BRAF-mutated melanoma cells by 50% (IC_{50}).

Cell line	IC_{50} PLX4032 (µM)
Colo-679	0.17 ± 0.03
Colo-679^{r}PLX4032$^{10\mu M}$	16.51 ± 1.31
IGR-39	0.23 ± 0.04
IGR-39^{r}PLX4032^{20}	9.94 ± 1.51
MelHO	0.13 ± 0.05
MelHO^{r}PLX4032$^{10\mu M}$	37.86 ± 2.82
RVH-421	0.10 ± 0.01
RVH-421^{r}PLX4032$^{10\mu M}$	14.61 ± 1.50
	PLX4720 (µM)
Colo-679	0.26 ± 0.07
Colo-679^{r}PLX4720$^{10\mu M}$	30.01 ± 1.95
IGR-39	0.41 ± 0.06
IGR-39^{r}PLX4720$^{20\mu M}$	23.93 ± 0.54
MelHO	0.21 ± 0.03
MelHO^{r}PLX4720$^{10\mu M}$	24.53 ± 7.35
RVH-421	0.05 ± 0.04
RVH-421^{r}PLX4720$^{10\mu M}$	37.71 ± 4.69

	IC_{50} vincristine (ng/mL)
Colo-679	3.57 ± 0.38
Colo-679^{r}VCR^{20}	80.59 ± 8.67
IGR-39	1.54 ± 0.10
IGR-39^{r}VCR^{10}	8.95 ± 2.23
MelHO	0.95 ± 0.09
MelHO^{r}VCR^{20}	95.77 ± 53.03
	IC_{50} mitoxantrone (ng/mL)
RVH-421	4.81 ± 0.53
RVH-421^{r}Mitox^{10}	24.97 ± 4.82
