# Supplementary material for: Association between acquired resistance to PLX4032 (vemurafenib) and ATP-binding cassette transporter expression
Source: BMC Res Notes. 2014 Oct 10;7:710. doi: 10.1186/1756-0500-7-710 (PMC4197243; doi:10.1186/1756-0500-7-710)
Supplement: Supplementary file 7 — Additional file 7: Table S6: A. Influence of the ABCB1 inhibitor verapamil (10 μM) on the sensitivity of ABCB1-expressing melanoma cells to PLX4720 or the cytotoxic ABCB1 substrate vincristine. Concentrations that reduce the cell viability by 50% (IC50) were determined after a five day incubation period by MTT assay. B. Influence of the ABCC1 inhibitor verapamil (10 μM) on the sensitivity of ABCC1-expressing melanoma cells to PLX4032, PLX4720, or the cytotoxic ABCC1 substrate vincristine. Concentrations that reduce the cell viability by 50% (IC50) were determined after a five day incubation period by MTT assay. C. Influence of ABCG2 inhibitor fumitremorgin C (2.5 μM) on the sensitivity of ABCG2-expressing melanoma cells to PLX4720 or the cytotoxic ABCG2 substrate mitoxantrone. Concentrations that reduce the cell viability by 50% (IC50) were determined after a five day incubation period by MTT assay. (PDF 14 KB) [file 13104_2014_3224_MOESM7_ESM.pdf]

**Suppl. Table 6A.** Influence of the ABCB1 inhibitor verapamil (10µM) on the sensitivity of ABCB1-expressing melanoma cells to PLX4720 or the cytotoxic ABCB1 substrate vincristine. Concentrations that reduce the cell viability by 50% ($IC_{50}$) were determined after a five day incubation period by MTT assay.

| | $IC_{50}$ (ng/ml) | cell viability in the presence of verapamil alone[1] | $IC_{50}$ in the presence of verapamil | fold sensitisation[2] |
|---|---|---|---|---|
| **Colo-679[r]VCR[10]** | | | | |
| vincristine | 81 ± 9 | 92 ± 16 | 3.3 ± 0.6[3] | 24.5 |
| **IGR-39[r]PLX4720[20µM]** | | | | |
| PLX4720 | 24 ± 0.5 | 90 ± 8 | 28 ± 1 | 0.9 |
| vincristine | 1.6 ± 0.1 | 90 ± 8 | 0.25 ± 0.01[3] | 6.4 |
| **MelHO[r]VCR[20]** | | | | |
| vincristine | 96 ± 53 | 88 ± 13 | 2.1 ± 0.4[3] | 45.7 |
| **RVH-421[r]PLX4720[20µM]** | | | | |
| PLX4720 | 38 ± 5 | 93 ± 5 | 35 ± 1 | 1.1 |
| vincristine | 0.76 ± 0.02 | 93 ± 5 | 0.17 ± 0.02[3] | 4.5 |

[1] values are % relative to non-treated control
[2] $IC_{50}$ drug/ $IC_{50}$ drug in the presence of verapamil
[3] $p < 0.05$ relative to vincristine or PLX4720 in the absence of verapamil

**Suppl. Table 6B.** Influence of the ABCC1 inhibitor verapamil (10µM) on the sensitivity of ABCC1-expressing melanoma cells to PLX4032, PLX4720, or the cytotoxic ABCC1 substrate vincristine. Concentrations that reduce the cell viability by 50% ($IC_{50}$) were determined after a five day incubation period by MTT assay.

| | **$IC_{50}$ (ng/ml)** | **cell viability in the presence of verapamil alone[1]** | **$IC_{50}$ in the presence of verapamil** | **fold sensitisation[2]** |
|---|---|---|---|---|
| **IGR[r]VCR[10]** | | | | |
| vincristine | 9.0 ± 2.2 | 95 ± 18 | 0.51 ± 0.06[3] | 17.6 |
| **IGR-39[r]PLX4032[20µM]** | | | | |
| PLX4032 | 9.9 ± 1.5 | 94 ± 7 | 12.0 ± 1.0 | 0.8 |
| vincristine | 1.5 ± 0.1 | 94 ± 7 | 0.11 ± 0.03[3] | 13.6 |
| **IGR-39[r]PLX4720[20µM]** | | | | |
| PLX4720 | 24 ± 0.5 | 90 ± 8 | 28 ± 1 | 0.9 |
| vincristine | 1.6 ± 0.1 | 90 ± 8 | 0.25 ± 0.01[3] | 6.4 |

[1] values are % relative to non-treated control
[2] $IC_{50}$ drug/ $IC_{50}$ drug in the presence of verapamil
[3] $p < 0.05$ relative to vincristine, PLX4032, or PLX4720 in the absence of verapamil

**Suppl. Table 6C.** Influence of ABCG2 inhibitor fumitremorgin C (2.5µM) on the sensitivity of ABCG2-expressing melanoma cells to PLX4720 or the cytotoxic ABCG2 substrate mitoxantrone. Concentrations that reduce the cell viability by 50% ($IC_{50}$) were determined after a five day incubation period by MTT assay.

| | $IC_{50}$ (ng/ml) | cell viability in the presence of fumitremorgin C alone[1] | $IC_{50}$ in the presence of fumitremorgin C | fold sensitisation[2] |
|---|---|---|---|---|
| **IGR$^r$VCR$^{10}$** | | | | |
| mitoxantrone | 6.2 ± 1.5 | 81 ± 12 | 1.6 ± 0.6[3] | 3.9 |
| **MelHO$^r$PLX4720$^{20µM}$** | | | | |
| PLX4720 | 25 ± 7.4 | 78 ± 15 | 23 ± 4.4 | 1.1 |
| mitoxantrone | 49 ± 3.5 | 78 ± 15 | 18 ± 2.5[3] | 2.7 |
| **RVH-421$^r$MITOX$^{20}$** | | | | |
| mitoxantrone | 25 ± 4.8 | 106 ± 10[4] | 6.3 ± 1.8[5] | 4.0[6] |
| **RVH-421$^r$PLX4720$^{20µM}$** | | | | |
| PLX4720 | 38 ± 4.7 | 102 ± 9[4] | 33 ± 8.0 | 1.2[6] |
| mitoxantrone | 174 ± 36 | 102 ± 9[4] | 29 ± 8.7[5] | 6.0[6] |

[1] values are % relative to non-treated control
[2] $IC_{50}$ drug/ $IC_{50}$ drug in the presence of fumitremorgin C
[3] $p < 0.05$ relative to mitoxantrone or PLX4720 in the absence of fumitremorgin C
[4] WK-X-34 (1µM) was used as ABCG2 inhibitor because fumitremorgin C caused toxicity in this cell line
[5] $p < 0.05$ relative to mitoxantrone or PLX4720 in the absence of WK-X-34
[6] $IC_{50}$ drug/ $IC_{50}$ drug in the presence of WK-X-34
