# Supplementary figures and images for: Association between acquired resistance to PLX4032 (vemurafenib) and ATP-binding cassette transporter expression
Source: BMC Res Notes. 2014 Oct 10;7:710. doi: 10.1186/1756-0500-7-710 (PMC4197243; doi:10.1186/1756-0500-7-710)

## Suppl. Figure 2

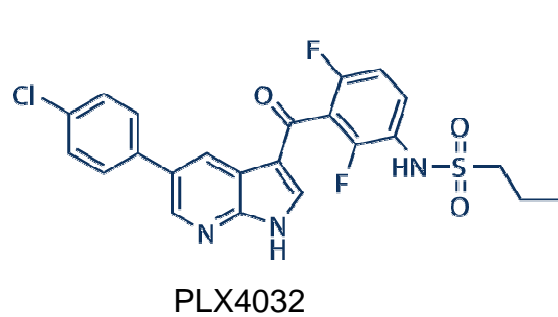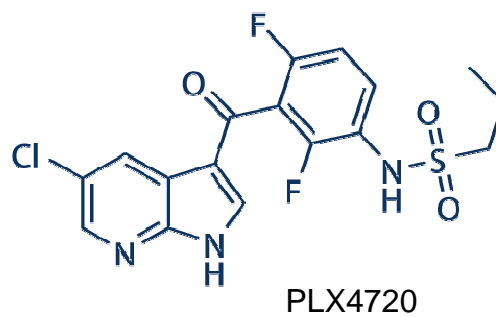

**Suppl. Table 2.** Chemical structures of PLX4032 and PLX4720.

Supplement: Supplementary file 8 — Additional file 8: Figure S2: Chemical structures of PLX4032 and PLX4720. (PDF 36 KB) [file 13104_2014_3224_MOESM8_ESM.pdf]
