# Supplementary material for: Association between acquired resistance to PLX4032 (vemurafenib) and ATP-binding cassette transporter expression
Source: BMC Res Notes. 2014 Oct 10;7:710. doi: 10.1186/1756-0500-7-710 (PMC4197243; doi:10.1186/1756-0500-7-710)
Supplement: Supplementary file 9 — Additional file 9: Figure S3: Potential clinical implications of the findings presented in this report. A) PLX4032 and PLX4720 interfere with ABCB1, ABCC1, and ABCG2 function. Since these ABC transporters are expressed at physiological barriers and control the absorption, body distribution, and excretion of their substrates, inhibition of these transporters may modify the pharmacokinetics of co-administered ABC transporter substrates including anti-cancer drugs and drugs that are administered for alternative indications (e.g. antihypertensive drugs). In addition, PLX4032 and PLX4720 may sensitise ABC transporter-expressing cancer cells to co-administered anti-cancer drugs that are ABC transporter substrates. B) Resistance formation to PLX4032 and PLX4720 may be associated with enhanced ABC transporter expression in cancer cells. This enhanced ABC transporter expression will result in cross-resistance of the PLX4032- or PLX4720-resistant cells to anti-cancer drugs that are substrates of the respective ABC transporters. (PDF 6 KB) [file 13104_2014_3224_MOESM9_ESM.pdf]

Suppl. Figure 3

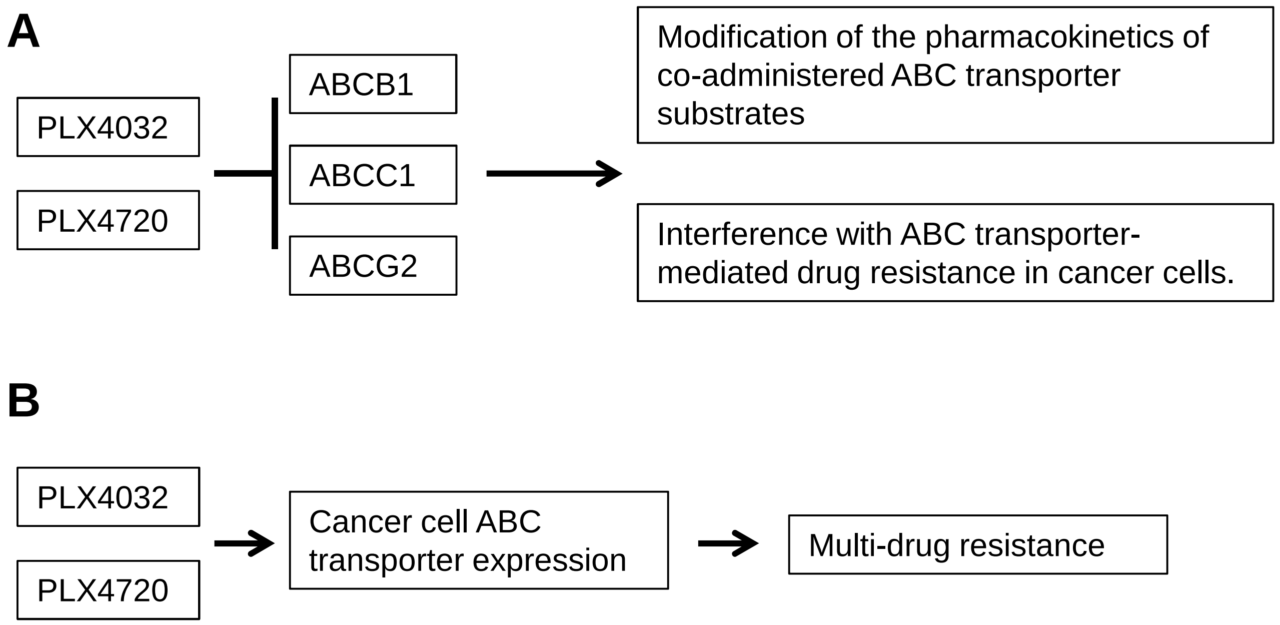

Suppl. Table 3. Potential clinical implications of the findings presented in this report. A) PLX4032 and PLX4720 interfere with ABCB1, ABCC1, and ABCG2 function. Since these ABC transporters are expressed at physiological barriers and control the absorption, body distribution, and excretion of their substrates, inhibition of these transporters may modify the pharmacokinetics of co-administered ABC transporter substrates including anti-cancer drugs and drugs that are administered for alternative indications (e.g. antihypertensive drugs). In addition, PLX4032 and PLX4720 may sensitise ABC transporter-expressing cancer cells to co-administered anti-cancer drugs that are ABC transporter substrates. B) Resistance formation to PLX4032 and PLX4720 may be associated with enhanced ABC transporter expression in cancer cells. This enhanced ABC transporter expression will result in cross-resistance of the PLX4032- or PLX4720-resistant cells to anti-cancer drugs that are substrates of the respective ABC transporters.
